# Development of Azole Resistance in *Aspergillus fumigatus* during Azole Therapy Associated with Change in Virulence

**DOI:** 10.1371/journal.pone.0010080

**Published:** 2010-04-09

**Authors:** Maiken Cavling Arendrup, Eleftheria Mavridou, Klaus Leth Mortensen, Eveline Snelders, Niels Frimodt-Møller, Humara Khan, Willem J. G. Melchers, Paul E. Verweij

**Affiliations:** 1 Unit of Mycology and Parasitology, Statens Serum Institut, Copenhagen, Denmark; 2 Department of Medical Microbiology, Radboud University Nijmegen Medical Centre, Nijmegen, The Netherlands; 3 National Centre for Antimicrobials and Infection Control, Statens Serum Institut, Copenhagen, Denmark; 4 Department of Internal Medicine, Sydvestjysk Sygehus, Esbjerg, Denmark; Duke University Medical Center, United States of America

## Abstract

Four sequential *Aspergillus fumigatus* isolates from a patient with chronic granulomatous disease (CGD) eventually failing azole-echinocandin combination therapy were investigated. The first two isolates (1 and 2) were susceptible to antifungal azoles, but increased itraconazole, voriconazole and posaconazole MICs were found for the last two isolates (3 and 4). Microsatellite typing showed that the 4 isolates were isogenic, suggesting that resistance had been acquired during azole treatment of the patient. An immunocompromised mouse model confirmed that the in vitro resistance corresponded with treatment failure. Mice challenged with the resistant isolate 4 failed to respond to posaconazole therapy, while those infected by susceptible isolate 2 responded. Posaconazole-anidulafungin combination therapy was effective in mice challenged with isolate 4. No mutations were found in the *Cyp51A* gene of the four isolates. However, expression experiments of the Cyp51A showed that the expression was increased in the resistant isolates, compared to the azole-susceptible isolates. The microscopic morphology of the four isolates was similar, but a clear alteration in radial growth and a significantly reduced growth rate of the resistant isolates on solid and in broth medium was observed compared to isolates 1 and 2 and to unrelated wild-type controls. In the mouse model the virulence of isolates 3 and 4 was reduced compared to the susceptible ones and to wild-type controls. For the first time, the acquisition of azole resistance despite azole-echinocandin combination therapy is described in a CGD patient and the resistance demonstrated to be directly associated with significant change of virulence.

## Introduction

*Aspergillus fumigatus* is the *Aspergillus* species involved in the vast majority of invasive infections. In contrast to *A. terreus*, which is intrinsically resistant to amphotericin B, and certain newly described species like *A. lentulus*, which are resistant to multiple antifungal drugs, *A. fumigatus* is normally susceptible to all three antifungal drug classes licensed for the treatment of invasive aspergillosis [1]. However, clinical failures involving *A. fumigatus* isolates with acquired triazole resistance are being increasingly reported over the recent years [2]–[7]. Although the impact of acquired resistance mechanisms on the fungus in terms of virulence and fitness is not yet understood, evidence is accumulating that isolates with acquired resistance are capable of causing aspergillus diseases, and that patients with azole resistant aspergillosis may fail to respond to azole therapy.

Two routes of resistance development have been proposed in *A. fumigatus*. The fungus may become resistant through exposure of the patient to azoles, which has been reported most frequently in patients with aspergilloma. It has been suggested that the mode of reproduction of the fungus is important for the phenotypic expression of azole resistance [8]. In patients with aspergilloma or other cavitary lesions, the fungus reproduces by asexual sporulation which facilitates transfer of resistance genes to spores and subsequent offspring [8]. Therefore, patients with cavitary lesions may be at increased risk of harbouring resistant fungus.

The other route of resistance development is exposure of *A. fumigatus* to azole fungicides in the environment [8]. *A. fumigatus*, being a saprophytic fungus, may be exposed to azole compounds in the environment and subsequent become cross-resistant to medical triazoles [5], [9]. This mode of transmission has been suggested in the Netherlands and *A. fumigatus* isolates resistant to medical triazoles were recovered from patients as well as from the environment [5]. The consequence of this mode of transmission is that possibly no specific risk group can be identified as patients will be randomly exposed to azole-susceptible and azole-resistant spores.

The most common mechanism of resistance in clinical isolates is a modification of the target site encoded by the *cyp51A* gene leading to reduced binding of the drug [2], [3], [10], [11]. Multiple point mutations have been reported and, although the phenotype depends on the specific amino acid alteration, resistance to multiple azoles is common [2], [3], [10], [12]–[14]. A specific L98H alteration in combination with a tandem repeat in the promoter region (designated TR/L98H) was found to be the dominant resistance mechanism in Dutch *A. fumigatus* isolates. It was shown that both alterations were required for the multi-azole resistant phenotype [15], and it is considered unlikely that both genomic changes could arise during azole therapy [4], [5], [15]. Finally, an increasing number of azole-resistant isolates are being reported that have no alterations in the *cyp51A* gene, indicating that other yet unknown mechanisms may play a role [3], [16].

Patients with chronic granulomatous disease (CGD) might be at risk for azole-resistant aspergillosis as they may receive life-long azole antifungal prophylaxis. Azole-resistant aspergillosis has been reported in two Dutch CGD patients [4]. In both patients the TR/L98H resistance mechanism was found in the recovered *A. fumigatus* isolates indicating that they had acquired the resistant isolate from the environment [5]. Azole prophylaxis in these patients may give the resistant spores a selective advantage to germinate and cause invasive disease compared to azole susceptible spores. Here, we report for the first time a CGD patient who developed azole resistance through prolonged combination treatment with azoles and echinocandin. The resistant phenotype was confirmed in an animal model and the mechanism demonstrated to be different than those previously described.

## Materials and Methods

### Origin of *A. fumigatus* isolates

Four isolates of *A. fumigatus* were obtained over a 2.5 year period from a 21 year-old patient with CGD at weeks 0, 108, 125, and 127 respectively (designated isolates 1 to 4) (Week 0 being the time point of isolation of study isolate no 1). Prior to the isolation of the study isolates the patient had suffered from several bacterial infections and *A. fumigatus* had been cultured from sputum 2 years earlier but this isolate was not stored. Pulmonary imaging showed mild emphysema with minor bullae in both lungs and progressive fibroses of the right lung with retraction and displacement of the mediastinum but no fungus ball containing cavities and no bullae related inflammation. *A. fumigatus* was thought to be responsible as this was the only pathogen repeatedly cultured from respiratory samples. He was treated with multiple courses of antifungal therapy. First course consisted of caspofungin monotherapy week −104 to −102, caspofungin + voriconazole combination therapy week −102 to −97, and voriconazole monotherapy week −97 to −96 with regression of pulmonary symptoms. Half a year later (week −67) the patient suffered from relapsing infection and due to the underlying immune defect and the presence of bronciectasis is was not possible to eliminate the fungus despite the following antifungal treatment regimens: voriconazole monotherapy week −67 to +12, caspofungin + voriconazole combination therapy week +12 to +90 followed by caspofungin + posaconazole combination therapy week +90 to +134.

Based on the radiologic appearance and the clinical course his disease could be classified as chronic fibrotic pulmonary aspergillosis [17]. The patient ultimately died from his pulmonary infection at week 134, autopsy was not performed.

Microbial samples from patients In Denmark are routinely submitted to the reference laboratory by hospitals. Only accredited scientists in the reference laboratory have access to these samples.

### Strain identification and susceptibility testing

Identification of the fungal isolates was based upon macroscopic and microscopic morphology and the ability to grow at 50°C. The identification was confirmed by sequencing of the β-tubulin gene, as described previously [18]. Genetic relationship of the four isolates was determined by microsatellite genotyping [5], [19]. Clinical *A. fumigatus* isolates AZN 8196 and v52–35 originating from patients with proven invasive aspergillosis were used as controls as well as *A. fumigatus* reference isolate NCPF2109.

In vitro susceptibility testing for the azoles was performed using the EUCAST method [20] and the M38A reference method of the Clinical Laboratory Standards Institute (CLSI) using a microbroth dilution format [21]. Susceptibility to caspofungin and anidulafungin was determined by Etest (AB Biodisk, Solna, Sweden) using RPMI-1640, 2% glucose agar plates (SSI Diagnostika, Hillerød,Herlev Hospital, Herlev, Denmark) and 2 days of incubation at 37°C. Aberrant growth in the inhibition zone was ignored.

### Growth kinetic assay

Growth kinetics on solid medium was performed using an inoculum of 0.5–2.5×10^4^ CFU/ml and a single spot (5 µl) on sabouraud and V8 juice agars in triplicates. Plates were incubated 4 days at 37°C and zone diameter measured after 24, 48, 72 and 96 hours. The average diameter was used to determine the radial growth rate (Kr). Kr was calculated using the linear regression of the radius versus time using a method described previously [22]. *In vitro* growth in fluent medium was determined using a previously described kinetic system [23], [24]. Briefly, 96-wells microtiter plates were inoculated with 10^4^ conidia, agitated for 15 s and incubated at 37°C inside a plate reader (Rosys Anthos ht3; Anthos Labtec Instruments GmbH, Salzburg, Austria) for 62 h. The optical density at 405 nm (OD_405_) was automatically recorded for each well every 10 min. The changes in OD over time were used to generate growth curves. In both assays the reference strain *A. fumigatus* NCPF2109 was included as comparator.

### Isolation of total RNA

The *A. fumigatus* isolates were adjusted to an inoculum of 10^7^ CFU and incubated 16 hours in 50 ml of Vogel's minimal medium at 37°C at 200 rpm in a 5% CO_2_ humidified chamber. The grown mycelia were recovered through attachment to 0.8 µm porous filter units by aspiration. The harvested mycelia were washed with 0.6 M MgSO_4_ and briefly air-dried at room temperature.

Dried mycelia specimens were placed in microcentrifuge tubes containing 0.6 mm glass beads and immediately frozen in liquid nitrogen. Homogenization was performed with MagNA Lyser Instrument. The fungal lysate was collected in extraction buffer (50 mM TrisHCl pH 8.0, 4% aminosalicyclic acid) and 1/5 volume acid-equilibrated phenol chloroform pH 4.7. Phase separation occurred according to the standard phenol-chloroform procedure: For optimal RNA yield, precipitation took place upon addition of 2 M LiCl, 1 mM EDTA and overnight incubation at 4°C. The precipitated RNA was centrifuged at 15000 x g at 4°C for 30 min, the supernatant was removed and the RNA pellet was collected in 300 µl of resuspension buffer (40 mM TrisHCl pH 7.5, 20 mM sodium acetate, 5 mM EDTA, 1% SDS). RNA samples were further dissolved at 37°C for 5 min and with an additional centrifuging for 5 min at 15000 x g remained debris were pelleted. The supernatant was transferred to a clean tube and 1/10 volume of 3 M sodium acetate pH 5.2 and 2.5 vol. of absolute alcohol was added. Samples were incubated at 4°C for 30 min and centrifuged at 15000 x g for 30 min at 4°C. The supernatant was removed and the pellet washed with 70% ethanol. RNA pellet was air-dried and RNA solubilization performed with RNAse-free water (autoclaved in the presence of 1% diethyl pyrocarbonate).

### RT-PCR for *Cyp51A* expression

The concentration and purity of the isolated total *RNA were determined* using a NanoDrop ND-1000 Spectrophotometer (Thermo scientific). First-strand cDNA synthesis was performed using 1 µg of RNA in the presence of 60 mM random hexamer primers. Thermocycling conditions were 10 min at room temperature; 60 min at 50°C and 5 min at 85°C. Cyp51A expression levels were determined by real time PCR using the LC480 instrument and the probes master kit (Roche applied Science). Thermocycling conditions were: 95x°C for 5 sec; and 50 cycles: 95x°C for 15 sec; 60°C for 45 sec and finally 1 time 40°C 30 sec. The sequences of primers used in the RT PCR were as follows: *Cyp51A* forward 5′-TCCTGCTCCTTAGTAGCCTGGTT -3′; *CYP51A* reverse 5′-GTGCTCCTTGCTTCACCTG -3′ and probe 6 -FAM-AGTGACAGCCCTCAGCGACGAA-BBQ. Actin was amplified by forward primer 5′-ATTGCTCCTCCTGAGCGTAAATAC-3′, reverse primer 5′-GAAGGACCGCTCTCGTCGTAC-3′ and probe 6-FAM-TCTGGCCTCTCTGTCCACCTTCCA-BBQ. Experimental samples were run in duplicate. Thus, all isolates were cultured twice, from each culture two separate cDNA amplifications were performed. In total four samples for each isolate were run by RT-PCR for cyp51A and Actin expression levels. The change in gene expression was determined using the ratio cyp51A/Actin. For each sample, a control containing only RNA was included. The background level detected for these controls was deducted from the corresponding samples.

### Immunosuppressed haematogenous invasive aspergillosis mouse model

The animal studies were conducted in accordance with the recommendations of the European community (Directive 86/609/EEC, 24 November 1986). All animal procedures were approved by the Danish Animal Experimentation Committee under the Ministry of Justice (number 2004/561-835). NMRI mice (weight 26–30 g, Harlan Scandinavia, Allerød, Denmark) were used throughout the study and immunosuppression was obtained through intraperitoneal administration of 200 mg/kg cyclophosphamide, at day −3 prior to inoculation and 100 mg/kg at day 0 (day of inoculation). Mice were challenged intravenously in volumes of 200 µl via a 25-gauge syringe.

### Virulence studies

A total of 30 NMRI mice, 5 isolates (the four different clinical isolates and a reference isolate (*A. fumigatus* NCPF2109)), 3 inocula per isolate, and 2 mice per inoculum was used. Thus a total of six mice were challenged with each isolate, two of which received a high inoculum (5×10^4^ CFU/ml), two an intermediate inoculum (10^4^ CFU/ml) and two a low inoculum (2×10^3^ CFU/ml).

### In vivo drug efficacy

A total of 121 NMRI mice (weight 26–30 g, Harlan Scandinavia, Allerød, Denmark) were immunosuppressed as described above. Eighty-one mice in groups of 6–15 were challenged on day 0 intravenously with conidia suspensions containing 1×10^5^ CFU *A. fumigatus* isolate 4, with an azole resistant phenotype, and 40 mice were infected with 1×10^4^ CFU/ml of the *A. fumigatus* isolate 2, with an azole susceptible phenotype in two separate experiments. A 10-fold difference in inoculum size between the S and the R isolate was chosen based upon the virulence studies in order to obtain similar mortality in the control groups. Mice were treated once daily on days 1–4 (Monday to Friday) and 7–10 (Monday to Thursday) with anidulafungin at a dose of 12 mg/kg intraperitoneally (i.p.), 20 mg/kg posaconazole p.o., a combination of the two antifungal drugs, or with glucose i.p. (control mice). Six mice per treatment group were sacrificed on days 4 and 8 and surviving mice were sacrificed on day 11. Kidneys were removed aseptically and stored at −80°C for subsequent CFU and quantitative PCR.

### Determination of fungal burden

Left and right kidney from each animal were homogenized using a Tissue Lyser (Qiagen, TissueLyser Type mm 301), UV radiated 2 metal beats (3 mm), and 30 HZ in 2 min. 100 µl of 10 fold dilutions (range undiluted – 1∶1000) of the tissue homogenate was used to inoculate Sabouraud agar and colonies were counted at day 2 for CFU determination.

Tissue samples were transferred to MagNA Lyser Green beads tubes (Roche Applied Science). TE buffer was added and homogenization was performed for 20 sec at 6500 rpm by using the MagNA Lyser instrument. Supernatant was used for DNA isolation by using the automated MagNA Pure system and the MagNA Pure LC Total Nucleic Acid Isolation Kit according to manufacturers protocol (Roche Applied Science). PhHV was added to all samples as an internal isolation control. Concentration of total isolated DNA was measured by using the Nanodrop ND-1000 Spectrophotometer (Thermo scientific). *Aspergillus* loads were determined by real time PCR using the LC480 instrument and the probes master kit (Roche applied Science). Thermocycling conditions were: 95x°C for 5 sec; and 50 cycles: 95x°C for 15 sec; 60°C for 45 sec and finally 1 time 40°C 30 sec. The 28S region of *Aspergillus sp*. was detected by using primers F 5′-TCCTGCTCCTTAGTAGCCTGGTT -3′, R 5′-GTGCTCCTTGCTTCACCTG -3′ and probe 6 -FAM-AGTGACAGCCCTCAGCGACGAA-BBQ. Addionally the PhHV isolation control was detected by using primers F 5′-TCCTGCTCCTTAGTAGCCTGGTT-3′, R 5′-GTGCTCCTTGCTTCACCTG -3′ and probe 6 -FAM-AGTGACAGCCCTCAGCGACGAA-BBQ. For the 28S detection a 8 fold dilution series of the cloned PCR product was included to calculate the number of copies per µl. The ratio of copies/µl and total DNA isolated ng/µl was calculated to determine the *Aspergillus sp.* load of each organ sample.

### Statistics

Survival among groups was compared using Mann-Whitney and Mantel-Cox tests. CFU-counts and quantitative PCR data among the various treatment groups were compared using the Mann-Whitney test.

## Results

### Strain identification and in vitro susceptibility

Following initial morphological identification, the *A. fumigatus* species of the four isolates was confirmed by sequence analysis of the highly conserved β-tubulin gene. Microsatellite genotyping showed that the four isolates were all STR*Af* type 3A: 48, 3B: 12, 3C: 15, 4A: 7, 4B: 8 and 4C: 10 and thus isogenic. The in vitro activity of antifungal agents against the four isolates is shown in Table 1. According to the recently proposed interpretative breakpoints [25], [26], isolate 1 and 2 were susceptible to itraconazole, posaconazole and voriconazole, while isolates 3 and 4 were resistant to these azoles. The antifungal activity of the polyenes and echinocandins was not different for the four isolates (Table 1).

**Table 1:** Susceptibility results obtained by CLSI/EUCAST microdilution (azoles) or by Etest (echinocandins) for four sequential isolates obtained from a CGD patient over a 127 week period.

Isolate no.	Week of collection	MIC (µg/ml)				
		Itraconazole	Voriconazole	Posaconazole	Anidulafungin*	Caspofungin*
1	0	0.125/0.5	0.5/1	0.016/0.125	0.004	0.064
2	108	0.25/0.5	0.5/1	0.031/0.125	0.004	0.064
3	125	>16/>4	4/>4	0.25/0.5	0.004	0.064
4	127	>16/>4	4/>4	0.25/1	0.004	0.125
Controls						
NCPF2109	NA	0.063/0.5	0.125/1	<0.016/0.125	0.004	0.064
TR/L98H	NA	>16/>4	8/>4	0.5/0.5	ND	0.25

*MICs are rounded to nearest upper two fold dilution value for the Etest endpoints.

NA: not applicable, ND: not done

### Development of secondary azole resistance following prolonged antifungal drug exposure

**Figure 1:**
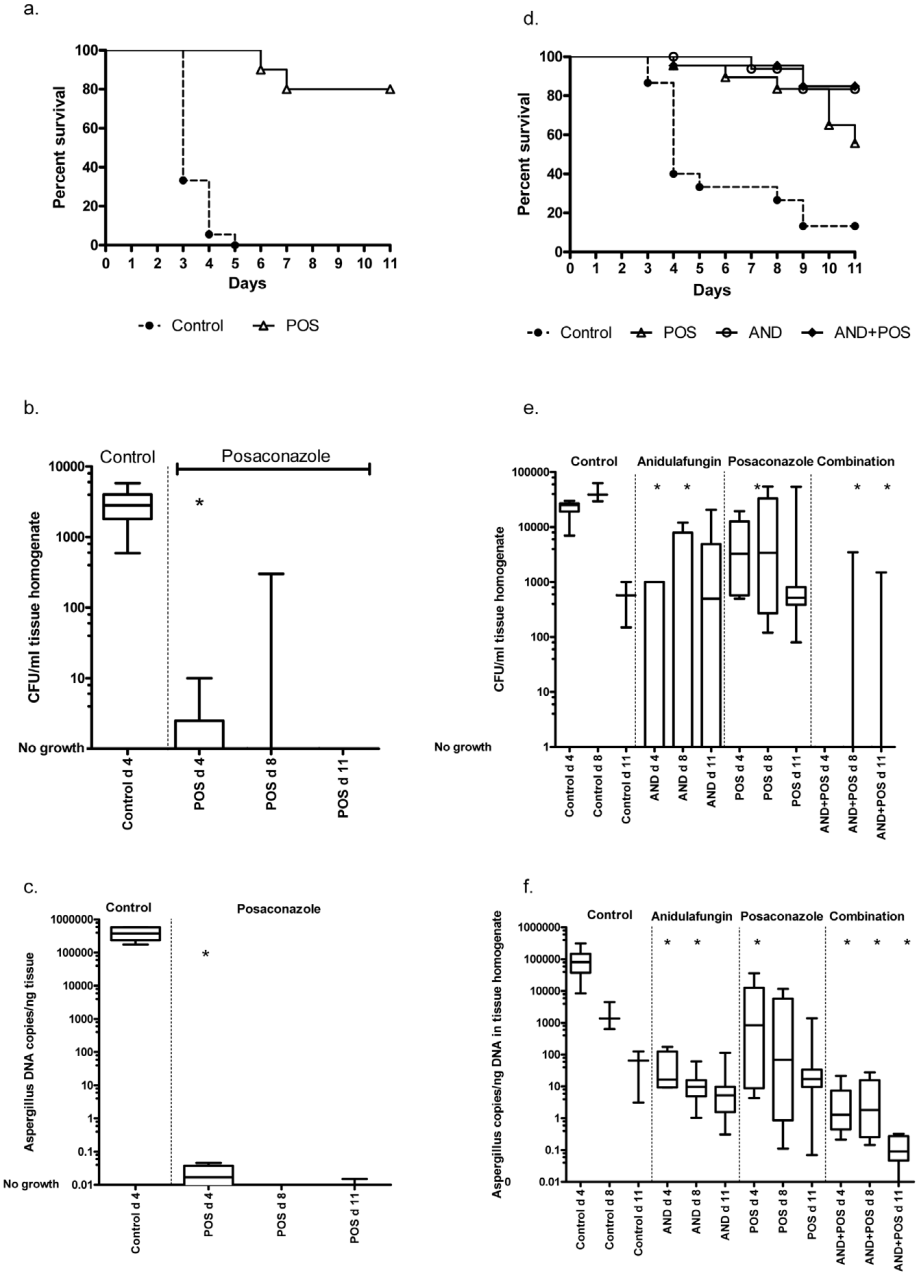
In vivo susceptibility of sequential *A. fumigatus* isolates from a CGD patient failing azole therapy. Susceptibility is shown as survival curve (a and d), as fungal CFU kidney burden (b and e) and as *Aspergillus* DNA load in kidney tissue. The early susceptible isolate (isolate no 2) is shown in fig. a, b and c and the late resistant isolate (isolate no 4) in fig. c, d and e. AND: anidulafungin, POS: posaconazole, AND+POS: combination therapy of anidulafungin and posaconazole, d4: day 4, d8: day 8, * P<0.05 compared to control.

The impact of the observed difference in vitro MICs was investigated in an immunocompromised murine model of disseminated aspergillosis. As the patient had been exposed to azole monotherapy as well as to azole + echinocandin combination therapy, both regimens were investigated. Posaconazole significantly improved survival, sterilised the kidneys and significantly reduced the kidney *Aspergillus* DNA copy number in animals challenged with susceptible isolate 2 when compared to untreated animals (Fig. 1a–c). However, posaconazole failed to significantly improve survival at the end of the study (Mann-Whitney P 0.0856) (fig. 1d), reduce kidney CFU day 8 and 11 (fig. 1e) and kidney *Aspergillus* copy number day 8 and 11 (fig. 1f) in animals challenged with isolate 4 and all posaconazole treated animals were kidney culture positive at all time points. In contrast, anidulafungin alone or in combination with posaconazole significantly improved survival (fig. 1d), significantly reduced kidney CFU day 4 and 8 (fig. 1e) and kidney *Aspergillus* copy number day 4, 8 and 11 for the combination (fig. 1f) and anidulafungin alone or in combination with posaconazole sterilised the kidneys in the majority of the animals. The posaconazole resistance in isolate 4 was not absolute, as significant kidney burden reduction was observed at the early time point day 4 and mortality was delayed in posaconazole treated animals (Mantel-Cox analysis P 0.0002) and as a tendency was seen towards better efficacy in the combination group than in the anidulafungin mono-therapy group.

### Mechanism of resistance

As several substitutions in the *cyp51A gene* were reported to be associated with an azole resistant phenotype [3], [26], we sequenced the coding *Cyp51A* gene together with the promoter region of this gene of all four isolates to identify the presence of potential mutations. Yet, the DNA sequencing results revealed no mutations neither in the coding gene nor in its 5′-upstream regulatory region. This suggested that another yet unknown mechanism was responsible for the change in azole activity.

**Figure 2:**
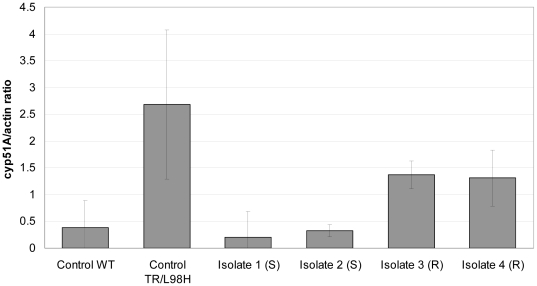
*CYP51A* mRNA levels in the 4 sequential *A. fumigatus* isolates and in a control wild type isolate AZN 8196 (WT) and a tri-azole resistant control isolate with over-expression of the CYP51A gene due to the L98H mutation and promoter tandem repeat.

In order to determine if the expression levels of the cyp51A was altered, we performed real time PCR experiments comparing the RNA expression levels of the four isolates and compared these to the levels observed in a non-isogenic susceptible wild type strain (AZN 8196), and a clinical CYP51A mutated isolate carrying the TR/L98H substitution (Figure 2). A 4 to 6-fold induction of the transcriptional profile of the resistant isolates 3 and 4 (0.2 (SEM 0.48) and 0.33 (SEM 0.11), respectively) compared to isolates 1 and 2 (1.4 (SEM 0.25) and 1.3 (SEM 0.52), respectively) and to the reference wild-type (0.38 (SEM 0.51)) was found. This increased level was half as high as the RNA expression level of the triazole resistant isolate with the TR/L98H mutation and the 34 bp tandem repeat in the *Cyp51A*-gene promoter (2.7 (SEM 1.4)).

### Impact of resistance on virulence

**Figure 3:**
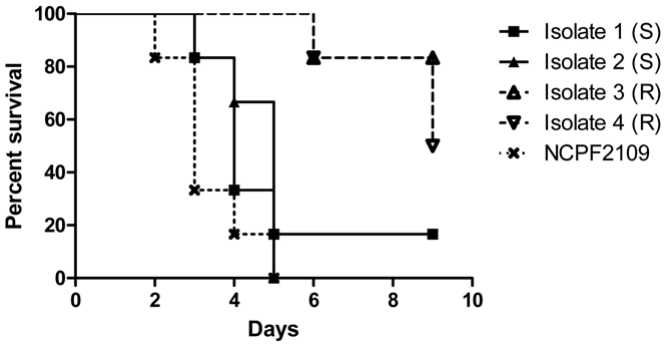
In vivo virulence of sequential isolates of *A. fumigatus* in immunosuppressed mice. Survival of mice in groups of six challenged with each of the four sequential isolates (1–4) or a control isolate (NCPF2109), respectively, is shown. The survival data was combined per isolate, thus each survival curve displays the mortality of six mice challenged with the indicated isolate, two of which were challenged with a high inoculum (5x10^4^ CFU/ml), two with an intermediate inoculum (10^4^ CFU/ml) and two with a low inoculum (2×10^3^ CFU/ml).

The virulence of the four isolates was investigated in the mouse model. As presented in Figure 3 there was a marked reduction of virulence by the resistant isolates 3 and 4 compared to the reference isolate, but also compared to the pre-treatment isolates 1 and 2 (Mantel-Cox test P values ranging between 0.0012 and 0.01). There was no significant difference in virulence between the susceptible isolates 1 and 2 (Mantel-Cox test P = 0.7) or between the resistant isolates 3 and 4 (Mantel-Cox test P = 0.29).

**Figure 4:**
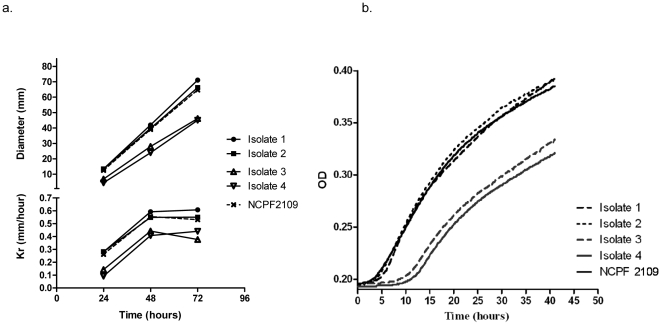
Growth rate of the 4 sequential isolates expressed as radial growth rate on solid V8 agar (a) and as optical densities in fluent medium (b). The laboratory strain NCP2109 is included as unrelated comparator. In the radial growth experiment (a) the growth rate is expressed in mean diameter (mm) and Kr (mm/hour) after 24–72 hours of two separate experiments performed in triplicate. In the kinetic evaluation of growth over time in liquid medium (b) growth is expressed as optical densities measured every 10 min.

Further investigations focused on the morphology of the isolates and the growth rates. Microscopic examinations of the conidia and mycelia morphology of all 4 serial strains revealed no obvious defects or changes. However, using *in vitro* growth analysis in duplicates on agar plates, the resistant isolates 3 an 4 showed a clear alteration in radial growth and a significantly reduced growth rate compared to the reference isolate and compared to the isolates 1 and 2 (Figure 4a). The kinetic evaluation of growth over time in liquid RPMI medium supported this finding as the first increase in optical density was delayed by 4 hours for isolates 3 and 4 and maximal growth was reduced compared with isolates 1 and 2 and the NCPF2109 control isolate (Figure 4b).

## Discussion

For the first time we show that azole resistance may develop in *A. fumigatus* during treatment of patients with CGD. As patients with CGD may receive antifungal prophylaxis for long periods of time, sometimes life-long, this is an important and clinically relevant observation. If *A. fumigatus* is cultured from CGD patients during azole prophylaxis the in vitro susceptibility profile should be determined. Selection of azole resistance during long term treatment has been described previously [2], [3], [6], [27], but in this case emerged despite concomitant caspofungin treatment. This is somewhat surprising as the sequential isolates were all in vitro susceptible to caspofungin and anidulafungin, and echinocandin susceptibility was confirmed in vivo in the mouse model. We have previously described disseminated breakthrough *A. fumigatus* infection following 40 days of caspofungin monotherapy in an immunocompetent ICU patient with no predisposing pulmonary disease [28]. These observations illustrate that in spite of low MEC values the non-cidal anti-aspergillus activity of echinocandins may not be sufficient to control aspergillus growth in vivo.

The set of isogenic isolates with different susceptibility profiles offered a unique opportunity to investigate the characteristics of resistance development in *A. fumigatus*. In clinical isolates azole resistance has been associated with impaired interaction between the drug and the target enzyme most commonly due to mutations at one of the hot spot regions at codons 54, 98 and 220 in the *cyp51A* gene [4], [10], [12], [13], [15], [29]–[31]. Also alteration at codon 138 (G138C) or codon 448 (G to S) has been detected in clinical isolates [2], [32] and association with resistance confirmed using laboratory generated mutants [33]. Finally, alterations at codons 216 (P to L), 431 (Y to C) and 434 (G to C) have recently been demonstrated in azole resistant isolates with no other resistance mutations but their association with resistance not yet experimentally confirmed [3]. It is likely that numerous mechanisms and target genes may be involved in development of azole resistance in *A. fumigatus*, of which the *Cyp51-*gene is the first that was identified. However, none of the above-mentioned mutations were found in our isolates, but the reduced susceptibility to triazoles was found to be associated with a 4–6 times increased *cyp51A* mRNA expression. Increased expression of the *Cyp51A* gene is commonly associated with the presence of transcriptional enhancers such as tandem repeats in the gene promoter region. Tandem repeats appear to be a common resistance mechanism in plant pathogenic moulds [8]. In environmental non-*Aspergillus* moulds, significant MIC increases of agricultural demethylase inhibitors have been associated with increased *cyp51A* expression in isolates without mutations in the target gene. A number of underlying mechanisms have been demonstrated 1) a 126 bp tandem repeat in the promoter region between nucleotide −672 and −43 in *Penicillium digitatum* leading to a 100 fold increase in expression level and with a proportional relationship between number of repeats and increase in MIC in laboratory generated mutants [34]; 2) upstream insertion of various truncated derivatives of a long interspersed nuclear element -like retrotransposon was detected in all 59 examined demethylase resistant isolates of *Blumeriella jaapii* [35], and 3) insertion of a unique 65 base pair repeat not normally present in the fungus was found in variable numbers in resistant isolates of *Monilinia fructicola* suggesting it is a mobile genetic element [36]. Finally, overexpression of the *cyp51* gene due to duplication of the entire chromosome containing the *CYP51* gene was observed in multi-azole resistant *C. glabrata*. A mechanism that was lost over repeated passages in drug free medium [37]. It was suggested that a tandem repeat may arise through sexual reproduction of the fungus, while point mutations may be associated with asexual reproduction [8]. Although we observed an increased expression of the *Cyp51A* gene in the azole-resistant isolates 3 and 4, compared to the isogenic isolates 1 and 2 with an azole-susceptible phenotype, this was not associated with the presence of a tandem repeat in the promoter region of the *Cyp51A* gene. Furthermore, we previously observed that recombinants with only the 34 bp tandem repeat showed increased expression of the *Cyp51A* gene, but this did not correspond with the full azole-resistant phenotype [15]. Only those isolates with the 34 bp tandem repeat and the substitution at codon 98 exhibited the multi-azole resistant phenotype [15]. Therefore, it remains unclear if the increased expression observed in our isolates is the cause of the resistant phenotype, although it may play a role. In order to detect or rule out other resistance mechanisms full genome sequencing of the susceptible and resistant isolates appears an appropriate way forward. As there was a limited time interval between culture of isolate 2 (azole-susceptible) and 3 (azole-resistant), comparative genomics may reveal other, yet unknown, resistance mechanisms.

The acquisition of azole resistance was associated with an important change of the virulence of the resistant isolates. In our murine model of invasive aspergillosis, the azole-resistant isolates were less virulent than the isogenic azole-susceptible ones and the wild-type controls. The in vivo virulence correlated with alteration of the growth rate of the resistant isolates. If these isolates remain capable of causing invasive disease is unclear although the aspergillus disease was thought to have contributed to the death of our patient and only resistant fungus was cultured from respiratory samples shortly before his death. Although the association between resistance and loss of fitness is well known in various microorganisms, to our knowledge this is the first time that it was observed in a human pathogenic mould. Loss of fitness may be associated with the underlying resistance mechanism. Preliminary experiments with *A. fumigatus* isolates with an azole-resistant phenotype due to mutations in the *Cyp51A*-gene, suggest that the virulence of these isolates is not reduced compared to wild-type isolates [38].

To our best knowledge this is the first report describing development of azole resistance during azole-echinocandin combination therapy and to describe an impact of acquisition of azole resistance on the virulence of the resistant isolates. A full genome comparison of initial and late isolates is in process in order to study the underlying mechanism in detail.
